# Complete biopsy-proven resolution of deposits in recurrent proliferative glomerulonephritis with monoclonal IgG deposits (PGNMIGD) following rituximab treatment in renal allograft

**DOI:** 10.1186/s12882-019-1239-8

**Published:** 2019-02-14

**Authors:** Jon Von Visger, Clarissa Cassol, Uday Nori, Gerardo Franco-Ahumada, Tibor Nadasdy, Anjali A. Satoskar

**Affiliations:** 1 Department of Internal Medicine, Division of Nephrology, Ohio State University Wexner Medical Center, Columbus, USA; 2 Department of Pathology, Division of Renal and Transplant Pathology, Ohio State University Wexner Medical Center, M018 Starling-Loving Hall, 320 W 10th Ave, Columbus, OH 43210 USA

**Keywords:** Proliferative glomerulonephritis with monoclonal IgG deposits, Anti-B-cell therapy, Renal allograft

## Abstract

**Background:**

Proliferative glomerulonephritis with monoclonal IgG deposits (PGNMIGD) is a disease entity classified under the group of “Monoclonal gammopathy-related kidney diseases”, and can recur after transplant. Clinical remission of proteinuria in patients with PGNMIGD has been previously shown following anti-B cell and/or anti-plasma cell therapies. Our case is the first to show complete histologic resolution of the glomerular monoclonal IgG kappa deposits in a case of recurrent PGNMIGD in renal allograft after rituximab and steroid treatment. This is a novel finding and it shows that the deposits are amenable to therapy. This case also highlights the importance of IgG subclass staining in the recognition of the monoclonal nature of the deposits. It is particularly important in PGNMIGD because only 20 to 30% of patients with this disease are reported to have detectable monoclonal gammopathy, and the deposits do not have any organized substructure on electron microscopic examination. Morphologically, they resemble polyclonal immune-type deposits seen in other immune complex glomerulonephritides such as lupus nephritis, infection-associated glomerulonephritis, and membranoproliferative glomerulonephritis (MPGN type I).

**Case presentation:**

The patient is a 44 year old Caucasian male who received a living unrelated donor kidney transplant for end-stage renal disease diagnosed 7 years before transplant. The reported native kidney biopsy diagnosis was membranoproliferative glomerulonephritis (MPGN) with IgG, C3 and kappa restricted deposits. Fourteen months post-transplant, he presented with abrupt worsening of graft function, proteinuria and serum IgG kappa monoclonal spike. Allograft biopsy was consistent with recurrent PGNMIGD, considering the native kidney diagnosis and interval post-transplant. He underwent plasmapheresis, IV pooled immune globulin, steroid pulse and taper, and anti-CD-20 Rituximab therapy. Patient had gradual decline in proteinuria and complete resolution of the immune deposits on repeat biopsy 3 months later. Unfortunately he subsequently developed chronic antibody-mediated rejection and transplant glomerulopathy and graft failure 34 months post-transplant.

**Conclusions:**

In a transplant setting, repeat allograft biopsies are frequently performed for graft dysfunction. This provides a good opportunity to study the evolution of the immune deposits following treatment. Our case shows complete histologic resolution of the deposits in allograft PGNMIGD.

## Background

Proliferative glomerulonephritis with monoclonal IgG deposits (PGNMIGD) is classified as one of the monoclonal gammopathy related kidney diseases [1–3]. Distinguishing features of PGNMIGD include – 1. Deposits are localized to glomeruli and are not seen in the tubules, interstitium or vasculature, unlike other monoclonal immunoglobulin-associated diseases such as amyloidosis or light/heavy chain deposition disease; 2. The deposits do not exhibit an organized substructure such as fibrils, microtubules or punctate granularity and therefore morphologically resemble polyclonal immune-type deposits; 3. A monoclonal spike in the serum or urine is identified in less than 30% of patients and overt hematologic malignancy is identified in less than 2 to 3% of the patients [1, 2]. The disease is reported to recur and can also develop de novo in renal allografts [4, 5] and presentation is reported to be similar to that in the native kidney, with nephrotic range proteinuria and rapid deterioration of graft function. Several case series have shown clinical remission and decrease in proteinuria after immunosuppressive therapy with Rituximab (with or without cyclophosphamide) in native and in transplant kidney [4, 6, 7]. Herein we describe the first reported case of recurrent PGNMIGD in renal allograft with complete resolution of the monoclonal IgG3 kappa deposits after Rituximab, steroid and plasmapheresis therapy demonstrated by serial allograft biopsies mapping the entire histologic course of the disease.

## Case presentation

Patient is a 44 year old Caucasian male who received a living unrelated donor kidney transplant at our institution. The diagnosis on the native kidney biopsy performed 7 years before transplant was membranoproliferative glomerulonephritis (MPGN) with IgG, C3 and kappa restricted deposits and patchy interstitial fibrosis. The patient was followed at an outside institution at the time and no specific therapy was provided for the disease in the native kidney prior to transplantation. The baseline post-perfusion allograft biopsy was unremarkable. The patient was maintained on mycophenolate and everolimus. By two months post-transplant, serum creatinine stabilized to 1.6 to 1.8 mg/dl for a year, and urine protein/creatinine ratio was less than 0.5 g/gram. Six months post-transplant, everolimus was changed to cyclosporine (due to arthralgias) with target levels of 600–1100 ng/ml for months 6 to 10 and thereafter reduced to 400 ng/ml.

**Fig. 1:**
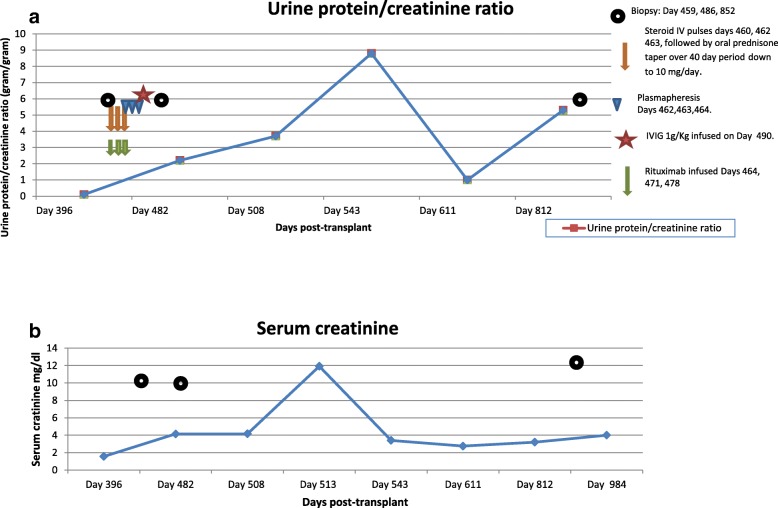
**a** Graph of patient’s post-transplant urine protein measurements at presentation, expressed as urine protein/creatinine ratios and days post-transplant. The timing of treatment with corticosteroids, Rituximab, plasmapheresis and intravenous immunoglobulin (IVIG) is shown. **b** Graph of patient’s post-transplant serum creatinine levels at presentation. Values during the period of graft dysfunction are shown

Fourteen months post-transplant, he presented with abrupt worsening of graft function, increasing proteinuria (Fig. 1a, b), active urine sediment and elevated rheumatoid factor (RF 1650 IU/ml), cryoglobulin test negative, requiring a kidney biopsy. Additionally he had IgG kappa monoclonal spike (214 mg/dl), serum free kappa light chains 189 mg/L (normal range 3.3–19.4), free lambda light chains 75 mg/L (normal range 5.7–26.3), kappa:lambda ratio of 2.5 (normal range 0.26 to 1.65), complements C3 126 (normal range 87–200 mg/dl), C4 38 (normal range 18–52 mg/dl).

### Biopsy 1 (15 months [day 459] post-transplant)

**Fig. 2:**
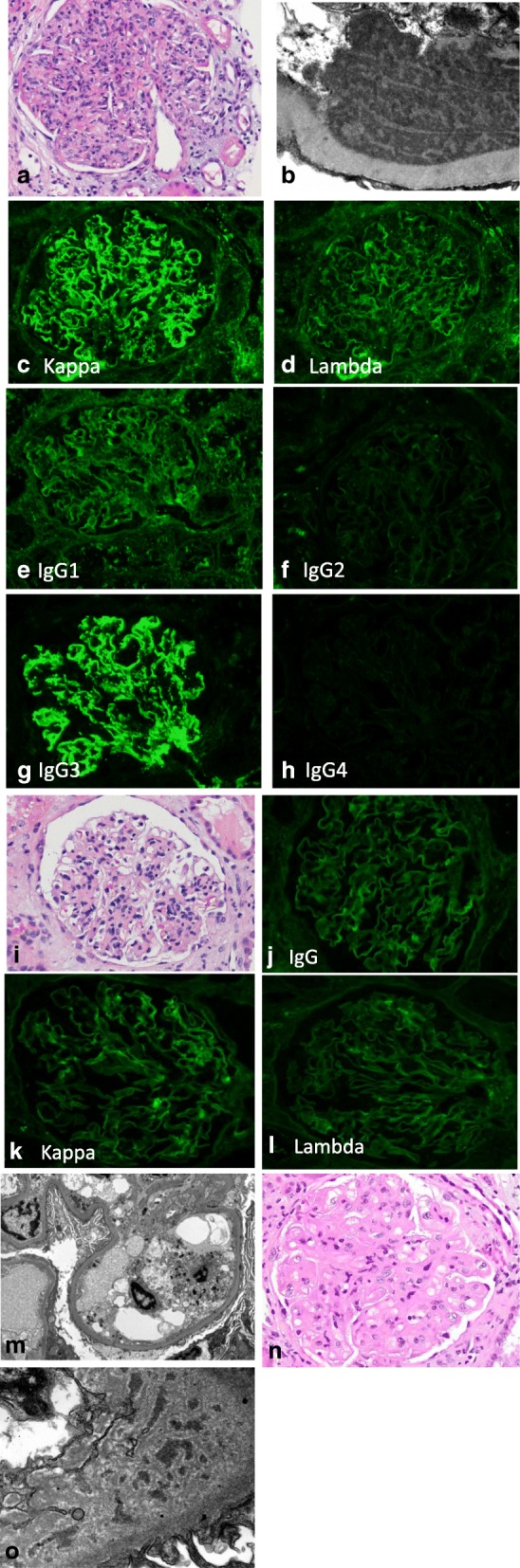
**a**-**h** Images from the first biopsy. **a** Endocapillary proliferative glomerulonephritis (H&E 400x). **b** Ultrastructural examination with electron dense immune-type deposits with lack of organized substructure (25,000x). **c** Direct immunofluorescence study shows bright granular IgG3. **d** Weak IgG1 **e** Negative IgG2. **f** Negative IgG4. **g** Bright kappa. **h** Negative lambda staining (all IF images 400x). **i**-**m** Images from second biopsy. **i** Mild mesangial expansion and hypercellularity (H&E 400x). **j** IgG staining shows absence of granular staining (400x). **k** Kappa trace staining. **l** Lambda negative (both 400x). **m** No electron dense deposits on ultrastructural examination (6000x). **n**-**o** Images from third biopsy. **n** Changes of transplant glomerulopathy (H&E 400x). **o** Ultrastructural examination with subendothelial widening, electron lucent amorphous material and entrapped non-specific electron densities (30,000x)

There were 18 enlarged glomeruli with diffuse endocapillary proliferative glomerulonephritis (Fig. 2a) with strong (3+) diffuse granular mesangial and capillary wall staining for C4d, IgG and kappa but no lambda (Fig. 2c, d), and corresponding electron dense immune-type deposits without any organized substructure (Fig. 2b). IgG subclass staining revealed strong staining for IgG3. Staining for IgG1, IgG2 and IgG4 was weak to negative (Fig. 2e-h). There was mild patchy interstitial inflammation, scattered tubules contained red blood cell casts. Interstitial fibrosis and tubular atrophy involved less than 20% of the renal cortex. Considering history of “MPGN” with kappa light chain restriction in the deposits in the native kidney, and the similar biopsy findings in the allograft with serum IgG kappa spike, a diagnosis of recurrent PGNMIGD was rendered.

### Treatment

He underwent plasmapheresis (3 sessions over 5 days) due to very high RF with concern for cryoglobulin and then anti-CD-20 Rituximab therapy (1000 mg weekly for 3 weeks), a single dose of IV pooled immune globulin (1 g/kg), steroid pulse and taper (750, 500, and 250 mg solumedrol per day, followed by oral steroid 1 mg/kg- 3 day taper by 5 mg increments), (Fig. 1). RF factor dropped to 137 within one week. Urine protein remained high and peaked at 8.7 g at 17 months post-transplant (requiring second biopsy) but decreased to 1 g at month 20. Serum monoclonal IgG kappa level dropped to 15.9 mg/dl. He developed CMV viremia (572 copies/ml), which responded to antiviral therapy.

### Biopsy 2 (17 months [day 486] post-transplant)

The endocapillary hypercellularity had largely resolved (Fig.2i). No glomerular IgG, kappa, lambda staining was seen on immunofluorescence (Fig. 2j, k, l). There was still prominent granular capillary wall and mesangial staining for C4d; however on ultrastructural examination, deposits were not seen (Fig. 2m). Interstitial fibrosis and tubular atrophy were mild, similar to that seen in the previous biopsy. Bone marrow biopsy was hypocellular but negative for lymphoma/myeloma.

### Follow-up

Unfortunately, the patient developed recurrent GI bleeding due to arterio-venous malformations and AKI requiring reduction in immunosuppression, and temporary hemodialysis for 2 months, with serum creatinine maintained at 2.7 mg/dl. Renewed proteinuria and rising creatinine were noted, month 27 post-transplant. Donor specific antibody was detected leading to the third biopsy.

### Biopsy 3 (28 months [day 852] post-transplant)

The glomeruli showed changes of transplant glomerulopathy (Fig.2 N) with mild peritubular capillary margination of inflammatory cells and diffuse peritubular capillary C4d staining. Moderate glomerular capillary wall and mesangial C4d staining were also observed. Ultrastructural examination showed subendothelial widening with electron lucent amorphous material and few entrapped non-specific electron densities (Fig. 2o). Immunofluorescence study showed mild focal smudgy glomerular IgG, IgA, IgM, kappa and lambda staining, representing non-specific trapping as in transplant glomerulopathy, but no discrete granular IgG deposits.

## Discussion and conclusions

PGNMIGD may be a consequence of a dysregulated clone of B-cells or plasma cells, without necessarily an obvious hematologic malignancy [2, 3, 8, 9]. The physicochemical properties of the monoclonal immunoglobulin (rather than high rate of production) make it prone to be deposited in the kidney causing nephrotoxicity (“monoclonal gammopathy of renal significance” [MGRS]) and therefore warrants clone-directed treatment even though it does not meet the criteria for overt myeloma or lymphoma. There is as yet no consensus on treatment regimens for PGNMIGD, but few case series have demonstrated clinical remission with anti-B cell drug Rituximab, along with cytotoxic agent cyclophosphamide and dexamethasone [6, 7]. The unique feature in our case is the demonstration of complete resolution of the monoclonal IgG deposits from the kidney after treatment, demonstrated by serial follow up biopsies. The proteinuria took longer to resolve but it did. It is plausible that podocyte injury takes longer to resolve.

Our case also demonstrates the utility of IgG subclass staining for accurate diagnosis of this disease [10]. Since the electron dense glomerular deposits of PGNMIGD morphologically resemble the immune complex deposits of other immune complex glomerulonephritides such as lupus nephritis, infection-associated glomerulonephritis, and membranoproliferative glomerulonephritis (MPGN) type 1 [1, 5], immunofluorescence staining for kappa and lambda light chains and more importantly for IgG subclasses is crucial for accurate diagnosis to demonstrate the monoclonal nature of the deposits. In some instances, both kappa and lambda staining may be seen and may not be sufficient to demonstrate monoclonality. This typically happens if additional heavy chains such as IgM (and their associated light chain) are also deposited or non-specifically entrapped in the deposits. In the absence of IgG subclass staining, these cases resemble MPGN type 1, and the disease may get incorrectly diagnosed and inadequately treated (as in our patient’s native kidney biopsy). Unfortunately the native kidney biopsy on our case was not available for review, but the biopsy report states “MPGN” with IgG kappa-restricted deposits. The patient received no specific treatment pre-transplantation and disease recurred in the allograft kidney within 2 years post-transplant. IgG subclass staining performed on the allograft biopsy at our institution clearly demonstrated the monoclonal IgG3 kappa deposits, clinching the diagnosis of recurrent PGNMIGD prompting appropriate anti-B cell therapy to be implemented immediately.

In spite of the resolution of immune complex deposition, as evidenced by lack of IgG and kappa staining on IF and absence of immune-type deposits on EM, proteinuria was slow to resolve. Although this phenomenon is difficult to explain, this is the usual course after Rituximab treatment of other proteinuric diseases as well, such as primary membranous glomerulonephritis. Proteinuria can take from 6 to 12 months to remit [11, 12]. Also, in the series by Guiard et al. [6], remission of nephrotic syndrome was seen after a mean delay of 9 months (range 4 to 24 months) after Rituximab initiation.

With the elevated rheumatoid factor and CMV infection in this patient, the possibility of infection-associated cryoglobulinemic glomerulonephritis emerged. But we want to emphasize that these are not cryoglobulin deposits because of several reasons: 1. Cryoglobulin test was negative, 2.no microtubular substructure in the deposits, 3. serum C3 and C4 complement levels were normal, 4. type II cryoglobulinemia is not usually associated with monoclonal IgG kappa deposits as seen in this case, 5. the predominant IgG subclass in cryoglobulins is reported to be IgG1 (not IgG3) [10].

There is experimental evidence showing resolution of glomerular immune complexes following cessation of antigenic stimulation [13, 14]. However, in clinical practice, repeat biopsies of the native kidney to look for resolution of glomerular deposits in immune complex glomerulonephritides are rarely performed (except for lupus flares), and patients are usually followed with clinical and laboratory parameters. Therefore it is difficult to study extent of resolution of immune complexes following treatment. In transplant setting however, repeat allograft biopsies are often clinically indicated and therefore provide an opportunity to study the evolution of glomerular deposits. The clinical course of PGNMIGD, like other kidney diseases, greatly depends on the degree of persistent proteinuria and the status of the underlying renal parenchyma. But in the transplant kidney, other complications such as graft rejections, infections, chronic allograft nephropathy, can also impact disease outcome. Our patient unfortunately developed chronic antibody-mediated rejection and transplant glomerulopathy, resulting in graft failure 34 months post-transplant and increasing proteinuria.

However, our case clearly demonstrates biopsy-proven resolution of the monoclonal IgG3 kappa deposits and marked decrease in the proliferative glomerular injury in PGNMIGD after Rituximab and steroid treatment. There was no relapse after cessation of anti-B cell treatment and alkylating therapy was not needed in this case. This is the first such documented case, demonstrating that the deposits in PGNMIGD are amenable to treatment.

## Abbreviations

CMV: Cytomegalovirus
EM: Electron microscopy
IF: Immunofluorescence
MGRS: Monoclonal gammopathy of renal significance
MPGN: Membranoproliferative glomerulonephritis
PGNMIGD: Proliferative glomerulonephritis with monoclonal immunoglobulin G deposits
RF: Rheumatoid factor
